# Measuring Relative Wind Speeds in Stratospheric Balloons with Cup Anemometers: The TASEC-Lab Mission

**DOI:** 10.3390/s22155575

**Published:** 2022-07-26

**Authors:** Daniel Alfonso-Corcuera, Mikel Ogueta-Gutiérrez, Alejandro Fernández-Soler, David González-Bárcena, Santiago Pindado

**Affiliations:** Instituto Universitario de Microgravedad “Ignacio Da Riva” (IDR/UPM), ETSI Aeronáutica y del Espacio, Universidad Politécnica de Madrid, Plaza. del Cardenal Cisneros 3, 28040 Madrid, Spain; mikel.ogueta@upm.es (M.O.-G.); alejandrojose.fernandez@upm.es (A.F.-S.); david.gonzalez@upm.es (D.G.-B.); santiago.pindado@upm.es (S.P.)

**Keywords:** cup anemometer, stratospheric balloon, wind speed, high-altitude calibration

## Abstract

This paper shows wind speed measurements from the TASEC-Lab experiment in a stratospheric balloon mission. The mission was launched in July 2021 from León (Spain) aerodrome. Measurements of horizontal wind speed in relation to the balloon gondola were successfully carried out with a cup anemometer. According to the available literature, this is the first time a cup anemometer has been used in a stratospheric balloon mission. The results indicate the need to consider the horizontal wind speed from the balloon ascent phase for thermal calculations of the mission.

## 1. Introduction

Wind speed measurement has numerous and varied applications today, ranging from the structural design of buildings and infrastructures [1] to the design and operation of wind power plants [2,3,4]. In this regard, the cup anemometer, invented by Robinson in the 19th century [5,6,7], constitutes one of the most widely used wind measurement instruments. Given the linearity of its transfer function, its easy calibration, its reliability, and its robustness [8], it is an ideal instrument to be used in the wind energy sector. Furthermore, it should also be noted that, regardless of the high precision of this instrument, a thorough calibration of a cup anemometer is required prior to installation [9].

Cup anemometers, on the other hand, have been used in environments other than the Earth’s surface, including space missions. Examples of these applications include the study of the use of a folding cup anemometer for a space mission to Mars by NASA’s Jet Propulsion Laboratory (JPL) in 1968 [10], or the use of this instrument at the Venera 9 and Venera 10 stations to measure the wind speed on the surface of Venus [11].

One of the most important advantages of the use of cup anemometers in low and very low-pressure environments is the possibility of extrapolating the results obtained in wind tunnel calibrations at standard pressure to the operation of anemometers at very low pressure, since the effect of Reynolds number scaling on the aerodynamic force normal to the cup is negligible [12,13].

Based on the above, previous work carried out at the *Instituto Universitario de Microgravedad “Ignacio Da Riva”* (IDR/UPM) at the *Universidad Politécnica de Madrid* (UPM) suggests that wind speed measurement technology based on 40 mm radius cup anemometers can be used to measure wind speed properly up to 25 km altitude [14].

This paper presents the cup anemometer as a plausible alternative to the sonic anemometer in balloon missions. Some examples of the use of sonic anemometers in these balloon missions have been found in the available literature, being in most cases commercial anemometers and missions whose maximum altitude reached was below the tropopause [15,16,17,18]. However, no example of the use of cup anemometers as wind speed sensors in high-altitude balloon missions have been found.

The use of sonic anemometers beyond the tropopause has specific problems related to the environmental conditions (very low temperatures and atmospheric pressure) that cause attenuation of sound wave propagation. Therefore, special anemometer calibration or even a new design need to be addressed if this instrument is selected for a high-altitude balloon mission.

Maruca et al. developed a very interesting experiment on the use of sonic anemometers at high altitudes [19]. They compared their data with two radiosondes launched the same day, and the results obtained from the sonic anemometer and the radiosondes seemed to be well correlated. They found, however, that the sonic anemometer started to have trouble taking measurements at approximately 17 km. At 18 km, the measurements were very commonly poor, and this is considered the top limit of the system. The sonic anemometer has also the advantage of being able to measure wind turbulence. Further research is needed in order to explore the limits of this instrument.

It is fair to say that cup anemometers also have specific problems, such as the poor speed of the dynamic response of the instrument [20,21], overspeeding [22,23] or the increase in the rotor’s shaft friction due to low temperatures [24]. Nevertheless, this is a very accurate and robust instrument, it is very well known (as it has been thoroughly studied during the last 150 years [25]), and its calibration is inexpensive. It is also worth mentioning that different solutions can be found in the literature to improve the dynamic response of the cup anemometer, from the proposal of low-inertia cup anemometers [26] to the development of signal-processing techniques, which address both the dynamic response and the overspeeding problems [27,28,29].

The use of a cup anemometer in fluids of different density was indirectly analyzed by Schubauer and Mason in 1937 [13]. These researchers studied the behavior of a “water current meter of the cup-wheel type” (hereinafter, flowmeter) operating in both a water stream and an air stream. In their paper, these researchers made some dimensional considerations to emphasize the importance of the dynamic pressure (in fact, they used the flow velocity multiplied by the square root of the density) on the performance of the flowmeter. After that, they showed the results of the flowmeter tests in both water and air flows, which support their statement.

The equation that describes the performance of a cup anemometer is the following:

(1) $$I\frac{d\omega }{dt}=Q_{A}-Q_{F}.$$

In the above equation, *I* is the moment of inertia of the rotor, *ω* is the rotational rate, *Q_A_* is the aerodynamic torque produced by the interaction of the rotating cups and the air flow, and *Q_F_* is the friction torque produced at the rotor’s shaft. If the friction forces are much lower than the aerodynamic ones, and the dimensional analysis is used, the performance of the anemometer (that is, the rotational rate, *ω*) is a function of a group of parameters and variables, such as the aforementioned moment of inertia, *I*, the dynamic pressure, the normal-to-the-cups aerodynamic force, *c_N_*, the Reynolds number, the cup radius, *R_c_*, the cups’ center rotation radius *R_rc_*, and a shape parameter of the rotor, Φ:

(2) ω=f1(12ρV2,cN,I,Re,Rc,Rrc,Φ).

Bearing in mind changes of the air flow density only, the rotation rate can be expressed as a function of the product of the flow velocity and the square root of the aforementioned flow density, and the Reynolds number:

(3) ω=f2(Vρ,Re).

If the work by Brevoort and Joyner [12] is taken into account, the variations of the Reynolds number have a reduced effect on the normal-to-the-cups aerodynamic force, *c_N_*, therefore:

(4) $$\omega =f_{3}\left(V\sqrt{\rho }\right).$$

In previous research [14], the use of a cup anemometer at high altitude above ground level was analyzed with an experimental campaign. To simulate the very low values of air density at high altitude, the rotation rate was studied in terms of the mentioned variable $V\sqrt{\rho }$. The performance was studied by taking into account the velocity at which the cup anemometer rotor stops rotating. It was concluded that a cup anemometer with a 40 mm cup radius could work properly at 25 km altitude.

### The TASEC-Lab Mission

In recent years, the use of stratospheric balloons by the scientific community has increased considerably, given the very competitive cost of this type of mission for many applications [30,31,32,33]. This increase in the use of stratospheric balloons has led to a greater need for further analysis of the thermal behavior in this type of mission, since it differs substantially from the thermal models used in space systems [34,35].

One of the main differences of the thermal model of a stratospheric balloon with respect to space platforms, such as satellites, is the thermal environment, which can be considered quasi-static and conditioned to the local characteristics of the area where the stratospheric balloon is located [36]. On the other hand, during the ascent phase of the stratospheric balloon, the convective heat transfer cannot be neglected, due to the low air temperature, the relative motion with respect to the wind, and the air pressure. During the float phase, this convection is natural, transferring heat between nearby elements.

In the ascent phase of a stratospheric balloon, in addition to natural convection, there is forced convection. Oscillations of the balloon gondola alter the wind flow pattern (i.e., they cause the existence of relative-to-the-gondola wind speeds), and therefore the heat transfer with the exterior. Such heat transfer can be critical in the tropopause environment (~11 km altitude), as non-negligible pressures (and, therefore, densities) are combined with high relative wind speeds with very low outside air temperatures [34].

**Figure 1:**
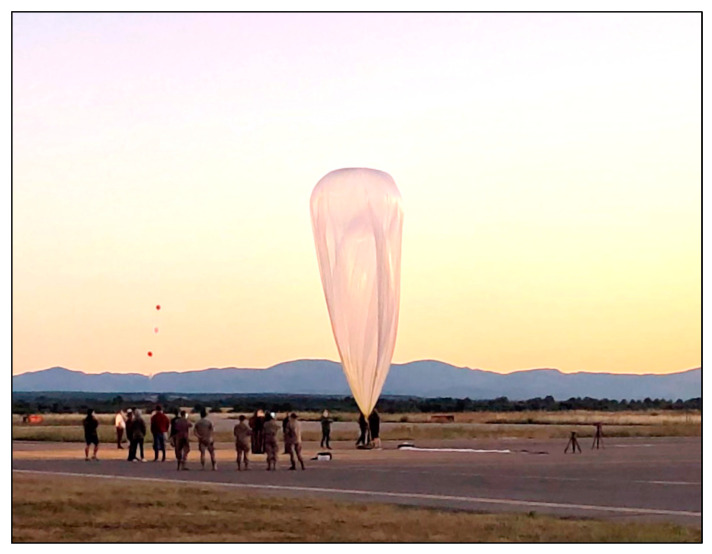
Launch of the TASEC-Lab mission from the León (Spain) aerodrome, on the morning of 16 July 2021.

The Thermal Analysis Support and Environment Characterization Laboratory (TASEC-Lab) is an experiment fully developed (i.e., designed, integrated and tested) at UPM by undergraduate, master’s, and doctoral students to analyze the convective heat transfer, thermal environment, and dynamic behavior of stratospheric balloons in their ascent and float phases. The experiment is designed following the CubeSat philosophy and using commercial off-the-shelf (COTS) components. The TASEC-Lab was launched aboard a stratospheric balloon (see Figure 1) from the aerodrome of León (Spain) on 16 July 2021 [37,38].

Due to the aforementioned characteristics of the TASEC-Lab mission, it was equipped with a cup anemometer to measure the wind speed in relation to the balloon gondola. This instrument was selected as a consequence of the results obtained previously in a wind tunnel at very low wind speeds [14].

The present paper describes the wind speed measurements carried out in the TASEC-Lab mission. Although the mission had to be aborted by the authorities before reaching the goal altitude of 25 km, interesting data were collected. Furthermore, and to the authors’ best knowledge, no other balloon mission has been equipped with a cup anemometer to measure the local wind speed. This paper is organized as follows: in Section 2, the experimental set-up is described, results are discussed in Section 3, and conclusions are summarized in Section 4.

## 2. Materials and Methods

**Figure 2:**
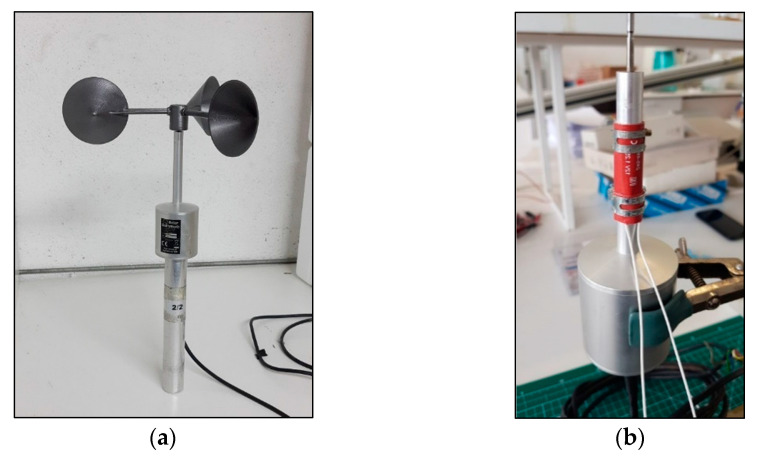
(**a**) TASEC-Lab cup anemometer. Rotor designed at the IDR/UPM Institute mounted on the body of the Vector Instruments A100 L2 cup anemometer; (**b**) Heater resistor and thermocouple installed on the body to preserve the temperature within the proper limits during the TASEC-Lab flight.

Taking into account the results of the very low speed tests, a rotor was developed whose characteristics are described in Table 1. This rotor was built by 3D printing in ABS material at the facilities of the IDR/UPM Institute. The rotor was coupled to the body of a First Class Vector Instruments A100L2 anemometer, whose characteristics are described in Table 2. Additionally, a heater was attached to the body of the cup anemometer to prevent loss of performance due to low atmospheric temperatures at high altitude levels over the ground [39]. Figure 2 shows the cup anemometer composed of the designed rotor, the Vector Instruments A100L2 sensor body, and the heater attached to the latter.

**Figure 3:**
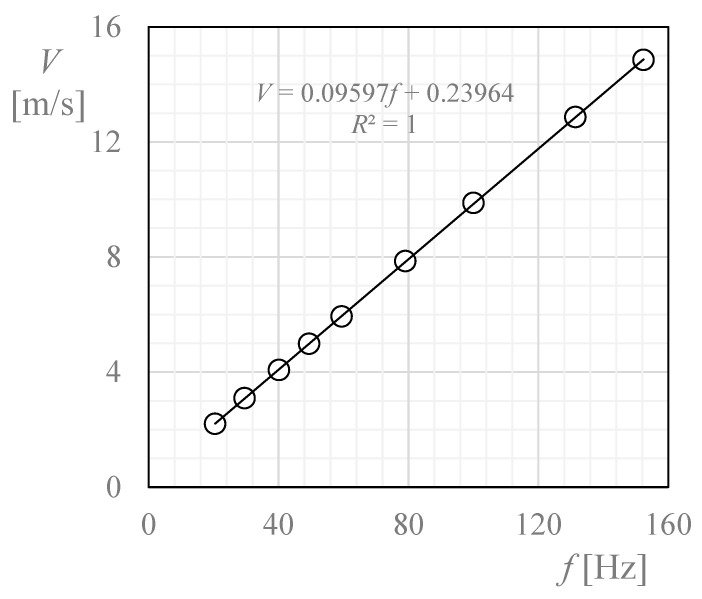
Transfer function (i.e., wind speed, *V*, vs. output frequency, *f*) of the TASEC-Lab cup anemometer measured at the LAC-IDR/UPM calibration lab.

The anemometer was calibrated in the S4 calibration tunnel of the LAC-IDR/UPM calibration lab, which is accredited according to UNE EN-ISO/IEC 17025. The facility consists of an open circuit wind tunnel with a test section that is 0.9 m wide by 0.9 m high. The wind tunnel is operated by 4 fans of 7.5 kW, with a flow uniformity below 0.2% in the test section [40]. The calibration of the anemometer was performed following the procedures established by MEASNET [41], according to IEC 61400-12-1 [42]. However, the normal calibration range, from 4 m/s to 16 m/s, was changed to reach lower wind speeds, from 2 m/s to 15 m/s, as low dynamic pressures are expected at high altitude above the ground [14]. Environmental conditions during the calibration process were 23.02 °C, 942.38 h Pa, and 36.8% humidity. The results of the calibration, that is, the transfer function (also called the calibration curve) [40]:

(5) V=Af+B,

is plotted in Figure 3.

**Figure 4:**
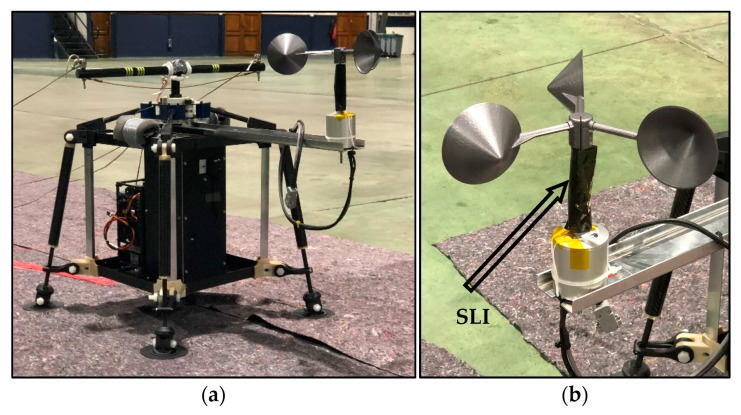
(**a**) Anemometer located on the upper side of the gondola; (**b**) SLI (Single-Layer Insulation) at the neck of the cup anemometer.

**Figure 5:**
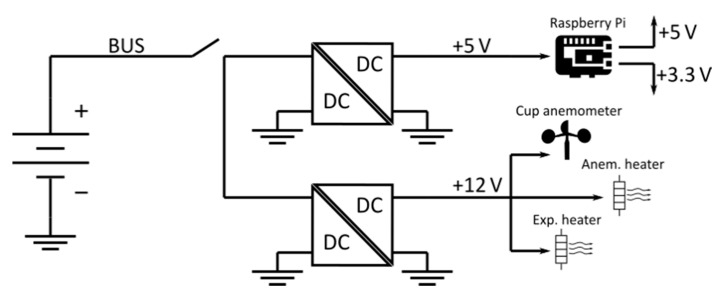
Power subsystem of the TASEC-Lab mission [38].

In the TASEC-Lab mission, the anemometer was placed on top of the gondola to measure the relative wind speed on the horizontal plane (see Figure 4). In addition, to limit the influence of extreme temperatures on the performance of the anemometer as much as possible, the neck of the anemometer (where the heater is located) was covered with black SLI (Single-Layer Insulation). The anemometer was powered at 12 V by a DC-DC converter connected to the TASEC-Lab battery; Figure 5 shows a sketch of the power subsystem of this mission.

A Raspberry Pi (RPi) model 3B+ computer with a Dual-Core Cortex-A53 (ARMv8) 64-bit SoC @ 1.4 GHz was used as the data acquisition system. The anemometer output was connected to one of the GPIO ports. This output signal was sampled at a rate of 3 kHz, as the number of pulses (one pulse was counted once the voltage of the output signal increased above 3.3 V) per second produced by the rotation of the instrument’s shaft was recorded each second [43]. More information on the optoelectronic system of the anemometer that produces the pulse train output signal can be found in [44,45]. The pulsecounting method was chosen to measure the pulse frequency instead of more sophisticated methods (i.e., FFT), due to the results of previous work [46].

## 3. Results and Discussion

**Figure 6:**
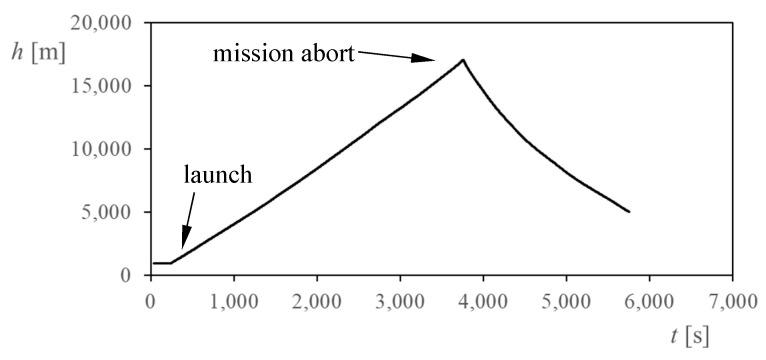
TASEC-Lab flight profile. Altitude, *h*, in relation to the mission time, *t*.

As previously mentioned, the TASEC-Lab mission was launched on 16 July 2021 from the León aerodrome (Spain). Unfortunately, due to unexpected delays from one of the partners, the mission had to be aborted after 1 h and 5 min by order of the Spanish air authority (AESA—*Agencia Estatal de Seguridad Aérea*), Figure 6 shows the altitude, *h*, reached by the balloon plotted in relation to the mission time, *t*. Therefore, only a relatively small amount of data from a more than 8-h mission is available. In the present paper, data from the ascent phase of the mission are analyzed as a relevant study of the performance of the cup anemometer at high altitudes above ground, which was suggested as a possibility in aforementioned work [14].

**Figure 7:**
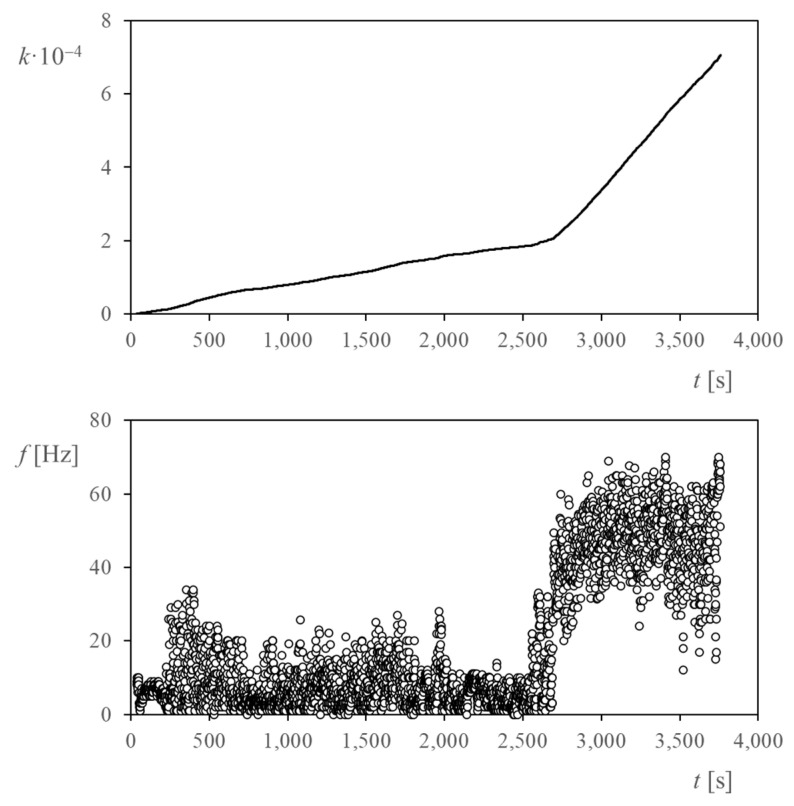
(**Top**) Evolution of the pulse count, *k*, with respect to altitude during the ascent phase of the TASEC-Lab flight. (**Bottom**) Cup anemometer frequency for each second of the ascent flight.

As described in the previous section, the following dataset was obtained during the TASEC-Lab mission: aggregate number of anemometer pulses, absolute atmospheric pressure, GPS altitude, and outside temperature. All values were measured and recorded at a frequency of 1 Hz. The records show that the anemometer remained operational during the entire ascent phase, as the pulse count, *k*, increased constantly throughout the flight; see Figure 7. The record of the number of pulses was translated into an output signal pulse frequency, *f*. Figure 7 shows this variable plotted in relation to the mission time, *t.*

It can be observed in this figure that the pulse count seems to be quite linear with time, with two clearly separated zones with different slopes. It seems to indicate a constant rotation rate of the anemometer in each of these zones of the ascent phase of the flight. This constant rotation rate is approximately confirmed by the frequency graph. However, this variable shows a great scatter, indicating quite large variations of the rotation rate caused by turbulence or oscillations of the gondola [33,47,48].

To derive the wind speed from the output frequency, it is important to:

- use the transfer function of the sensor (i.e., the calibration constants, A and B; see Equation (5) and Figure 3 to obtain the wind speed at ground level, *V_ref_*, in relation to the output frequency, *f*, and then to
- translate that wind speed at ground level into a local wind speed (i.e., at the proper altitude), *V*.

The mathematical equation that gives the value of the local wind speed, *V*, is the following [14]:

(6) $$V=V_{ref}\sqrt{\frac{\rho_{ref}}{\rho }}=\left(Af+B\right)\sqrt{\frac{\rho_{ref}}{\rho }}.$$

In the above equation, *ρ_ref_* and *ρ* refer to the air density values at ground level (where the calibration was carried out) and at the proper altitude, respectively. The absolute pressure values converted to altitude based on the ISA model and adjusted by means of the data provided by the GPS were used to obtain the values of *ρ*.

**Figure 8:**
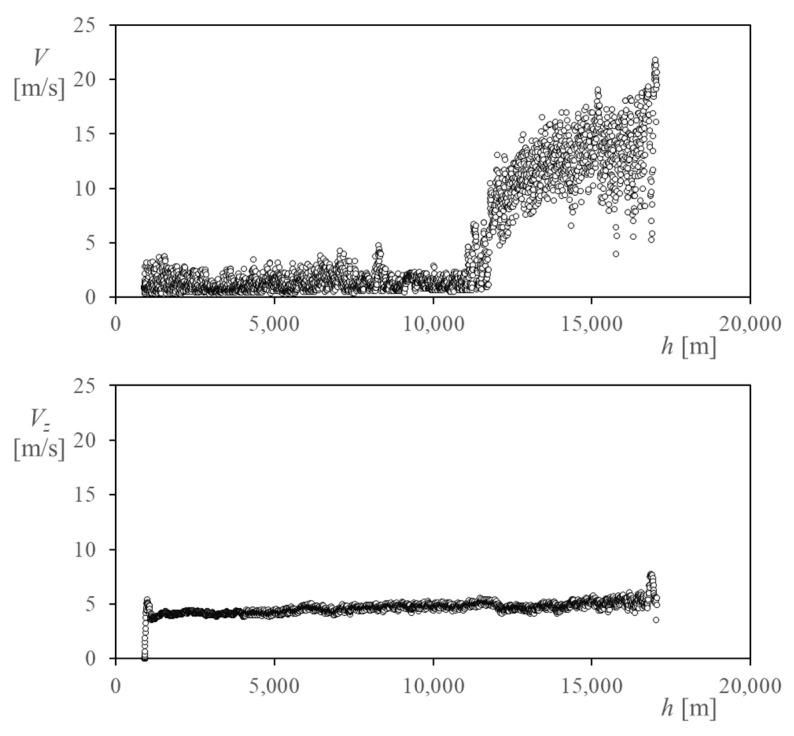
(**Top**) Horizontal wind speed measured by the cup anemometer, *V*, in relation to the altitude, *h*, along the ascent phase of the TASEC-Lab flight. (**Bottom**) Vertical speed (climb speed) of the gondola, *V_z_*.

The wind speed measured by the cup anemometer, *V*, in relation to the altitude, *h*, is shown in Figure 8. Additionally, the vertical speed (or climb speed) of the gondola/balloon, *V_z_*, is also plotted in the bottom graph of the figure. The almost constant vertical velocity (4–5 m/s) described in this second graph is consistent with the relatively constant climbing rate shown in the graph of Figure 6. This result is also described by other authors in recent works [33,49].

**Figure 9:**
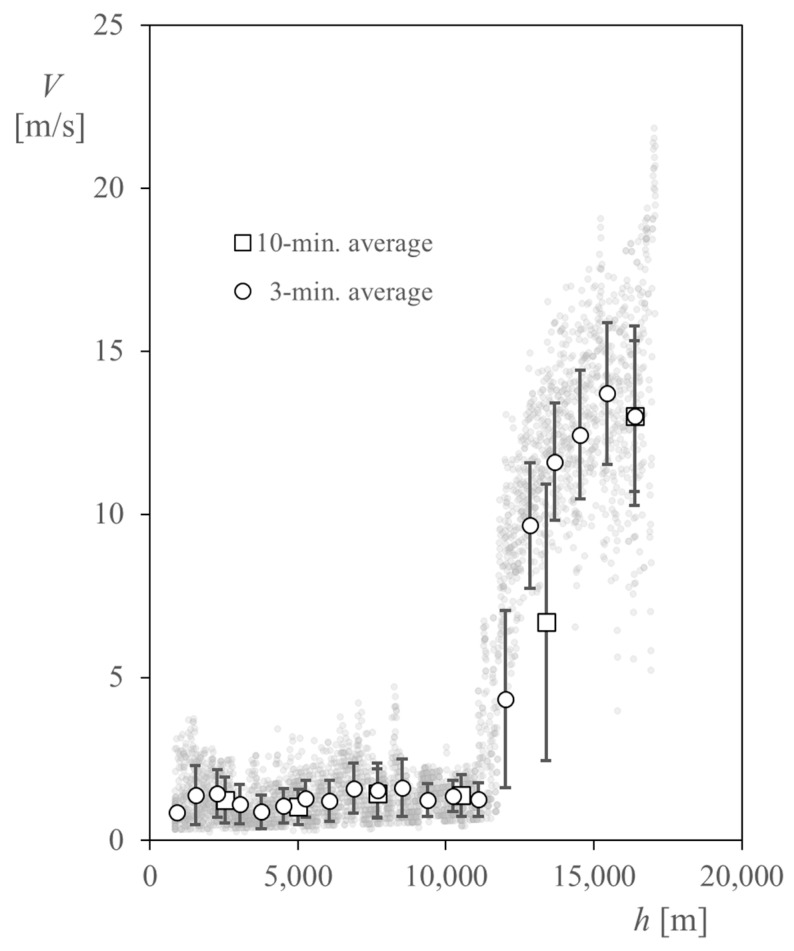
Horizontal wind speed measured by the cup anemometer, *V*, in relation to altitude, *h*, along the ascent phase of the TASEC-Lab flight. The 3-min. and 10-min. average values (calculated before the corresponding altitude) have been included, together with the standard deviation bars (see also Table 3 and Table 4).

Regarding the horizontal wind speed, *V*, shown in Figure 8, the results show quite large values beyond the tropopause (located at around 12 km altitude), according to the data and the wind description from [33,47,48]. The 3-min (3-min. hereinafter) and 10-min (10-min. hereinafter) average values of the horizontal wind speed have been plotted in Figure 9 over the recorded values of this variable. In addition, standard deviation bars have been added to these values (see also Table 3 and Table 4). As stated previously, larger horizontal wind speeds are measured within the stratosphere compared to those within the troposphere, also with larger scatter values. The larger values of the wind speed deviation correspond to the tropopause, due to the shear stress between the troposphere and the stratosphere, as expected. Additionally, the 3-min. average values of the vertical wind speed have been added to Table 3.

According to the measurements of horizontal relative speed, it is clear that during the first stage of the ascent phase, the climb speed has a greater magnitude than the horizontal relative wind speed and, therefore, the convective effects due to the relative speed of the system are dominated by this climb speed. However, at the tropopause, by means of the first instants of the second stage of the ascent phase, it is shown that there is a transition where the domination of the convective effects changes, since from that altitude the magnitude of the horizontal speed is greater (even twice or three times the climb rate).

This fact is relevant due to the traditional thermal design of the types of platforms carried aboard stratospheric balloons, which takes into account only the worst scenarios from the thermal point of view in the float phase [50]. Traditionally, thermal analyses of stratospheric balloons considered only the float phase to define the worst scenarios. However, according to the data obtained in the TASEC-Lab mission, as a consequence of the combination of low air temperatures and high horizontal relative speed, the ascent phase should also be considered (or at least its effects analyzed) in order to reach more accurate estimations of the cold case of the whole mission.

As an example of the importance of horizontal velocity, the convection coefficient, *h_c_*, of a vertical aluminum square plate was analyzed using the measured data. It was assumed that one of its faces was insulated, while the other was exposed to both convection and thermal radiation. The length of the plate sides was set to 0.1 m, with a thickness of 0.005 m. The optical coating is set with *α/ε* = 0.3. The power dissipation of the plate was set to 100 W/m^2^.

Two different cases were studied. In the first case, only the vertical velocity, *V_z_*, was considered, while in the second one, both the vertical and horizontal velocities, *V_z_* and *V*, were taken into account (see Figure 8). ISA atmosphere and thermal radiative loads are defined according to [51,52].

**Figure 10:**
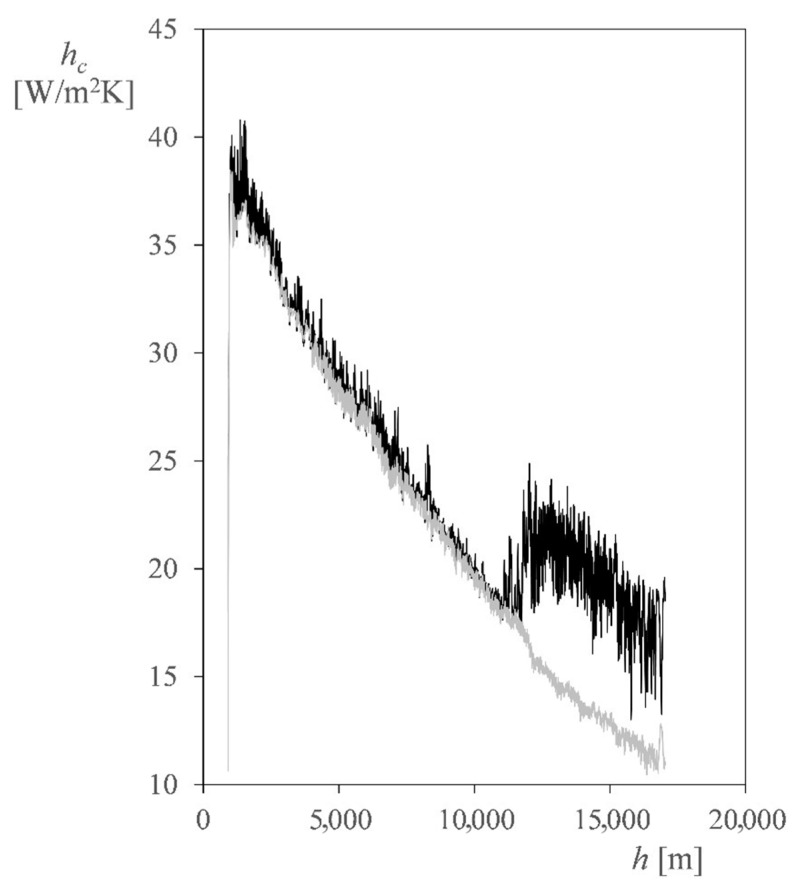
Convection coefficient, *h_c_*, of a vertical aluminum square plate that is 0.1 m side-long and 0.005 m thick vs. altitude. This coefficient was calculated with the wind speed measurements. The black line represents the case considering both vertical and horizontal wind speeds, *V_z_* and *V*, and the grey line represents the case considering only vertical wind speed (see Figure 8).

The results in Figure 10 clearly show that, at the tropopause, where the horizontal speed has a change in magnitude, the convection coefficient also increases. Thus, from the thermal point of view, the horizontal speed has to be taken into account during the ascent phase.

Using the GPS data of the balloon, two analysis processes have been performed to validate the results.

**Figure 11:**
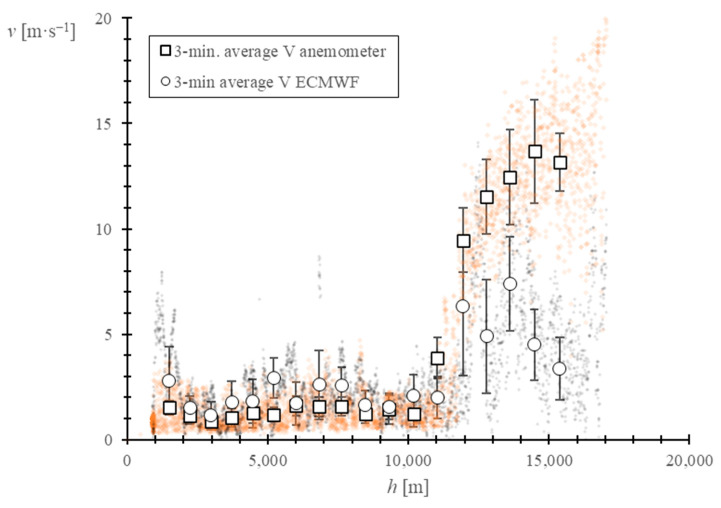
Evolution of the ECMWF estimated relative wind speed and measured wind speed vs. altitude. The relative wind speed estimation is obtained by adjusting the absolute wind speed estimations from the ECMWF ERA5 database with speed data from the balloon GPS. The black dots represent the relative wind speed estimation and the orange dots represent the measured wind speed. The 3-min. average, together with the standard deviation, are shown for both variables.

First, using the ECMWF Copernicus Program ERA5 reanalysis dataset [53], combined with the GPS speed data, an estimation of the relative horizontal wind speed was obtained. A comparison with the obtained results can be found in Figure 11. As it can be observed, results from this validation method do not seem to be conclusive, as the estimated values differ significantly with the measured ones. Nevertheless, the relative wind speed estimation shows higher wind speeds above the tropopause, which is consistent with the measured data. The low resolution of the estimations of the ERA5 dataset in terms of time, space, and pressure level (see Table 5) may be the cause of this deviation.

Then, the balloon GPS data were used to derive the horizontal acceleration of the balloon, which, as a function of the drag force caused by the surrounding wind, can be considered dependent on the horizontal relative wind and air density [54]:

(7) $$a=f_{4}\left(\rho V^{2},C_{D}\right).$$

Considering the drag coefficient, *C_D_*_,_ to be constant during the flight:

(8) $$a=f_{5}\left(\rho V^{2}\right).$$

And taking into account Equations (4) and (5):

(9) $$\omega^{2}=f_{6}\left(\rho V^{2}\right).$$

**Figure 12:**
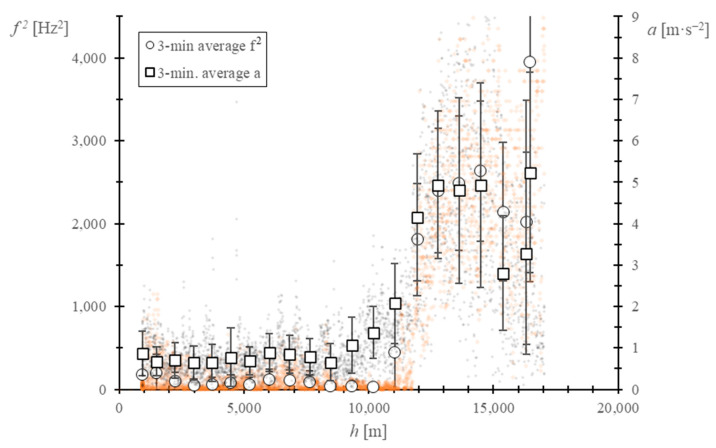
Evolution of the square of the frequency of the anemometer’s output signal and the horizontal acceleration module vs. the altitude of the balloon. The acceleration is calculated with the balloon’s GPS position data. The black dots represent the horizontal acceleration and the orange dots the square of the output signal frequency. The 3-min. average, together with the standard deviation, are shown for both variables.

From the equations above, it can be concluded that the horizontal acceleration module should be qualitatively correlated with the square of the output signal frequency. The comparison between both variables can be found in Figure 12. A clear relationship is found, with a very similar evolution of the variables. Above the tropopause, a high increase can be observed together with an increase in the standard deviation of the data. Results from this analysis seem to be coherent and indicate that the data obtained from the anemometer at the tropopause correspond to the horizontal component of the wind.

## 4. Conclusions

In the present paper, the results of the wind speed measurements from the TASEC-Lab experiment are described. This thermal analysis experiment was carried out in a stratospheric balloon mission, launched in July 2021. The horizontal wind speed in relation to the balloon gondola was measured using a cup anemometer. The most relevant conclusions of this paper are as follows:

- It is possible to use cup anemometers in stratospheric balloon missions. This instrument is an adequate alternative to the sonic anemometer, whose measurements can be affected by the low air densities at high altitudes above ground.
- Large wind speeds (up to 22 m/s) were measured beyond the tropopause, the deviation of these measurements being much larger than those of the troposphere.
- The highest variations of the horizontal wind speed were measured at the tropopause (at 12 km altitude), as expected.

The results seem to suggest the need to include the horizontal wind speed in heat transfer calculations within the balloon ascent phase, as it is of the same order of magnitude as the vertical wind speed (that is, the ascent velocity of the balloon’s gondola).

## Figures and Tables

**Table 1:** Characteristics of the rotor used for the TASEC-Lab mission.

**Cup radius, *R_c_* [mm]**	40
**Cup diameter, *D_c_* [mm]**	80
**Cup center rotation radius, *R_rc_* [mm]**	98.5
**Material**	Acrylonitrile Butadiene Styrene (ABS)

**Table 2:** Characteristics of the anemometer used for the TASEC-Lab mission.

**Supply voltage, *V_s_* [V]**	12
**Rotor speed measurement**	By interruption of optical beam
**Pulse output voltage, *V_o_* [V]**	5
**Number of pulses per rotor revolution, *N_p_***	25 (disk type K)
**Pulse rise/fall time [μs]**	25, duty cycle 50% (±25%)
**Operating temperature range**	−30 °C to 70 °C

**Table 3:** 3-min. average horizontal and vertical wind speeds, *V*_3_ and *V_z_*_3_, and standard deviations, *σ_V_*_3_ and *σ_Vz_*_3_.These values were calculated in the 3-min. lapse before the corresponding altitude).

*h* [m]	*V*_3_	*σ_V_*_3_	*V_z_*_3_	*σ_Vz_*_3_
913	0.86	0.17	−0.02	0.10
1537	1.39	0.90	3.36	1.49
2276	1.44	0.73	4.10	0.13
3029	1.11	0.60	4.18	0.14
3761	0.88	0.51	4.06	0.09
4513	1.06	0.52	4.18	0.12
5264	1.28	0.57	4.17	0.13
6062	1.22	0.63	4.43	0.19
6873	1.60	0.77	4.51	0.18
7681	1.54	0.83	4.49	0.20
8516	1.62	0.87	4.63	0.14
9373	1.25	0.50	4.76	0.17
10,235	1.37	0.48	4.79	0.15
11,095	1.26	0.52	4.78	0.15
12,004	4.34	2.72	5.05	0.15
12,835	9.65	1.92	4.62	0.17
13,673	11.61	1.80	4.65	0.21
14,534	12.44	1.98	4.79	0.24
15,445	13.71	2.17	5.05	0.25
16,373	13.02	2.76	5.16	0.33

**Table 4:** 10-min. average horizontal wind speed, *V*_10_, and standard deviation, *σ_V_*_10_. These values were calculated in the 10-min. lapse before the corresponding altitude).

*h* [m]	*V*_10_	*σ_V_*_10_
2533	1.24	0.71
5010	1.03	0.55
7681	1.44	0.75
10,525	1.38	0.65
13,383	6.69	4.24
16,373	13.01	2.32

**Table 5:** Characteristics of the ERA5 dataset used to obtain the estimated wind speed [53].

**Model version**	IFS Cycle 41r2
**Assimilation system**	IFS Cycle 41r2 4D-Var
**Horizontal spatial resolution**	31 km
**Vertical spatial resolution: Pressure levels [h Pa]**	100; 125; 150; 175; 200; 225; 250; 300; 350; 400; 450; 500; 550; 600; 650; 700; 750; 775; 800; 825; 850; 875; 900; 925; 950
**Temporal resolution**	1 h

## Data Availability

The data used in this research are owned by *Instituto Universitario de Microgravedad “Ignacio Da Riva” (IDR/UPM), Universidad Politécnica de Madrid*. Any request regarding the data should be directed to this institution.
